# Association of Hematological Variables with Team-Sport Specific Fitness Performance

**DOI:** 10.1371/journal.pone.0144446

**Published:** 2015-12-07

**Authors:** Franck Brocherie, Grégoire P. Millet, Anna Hauser, Thomas Steiner, Jon P. Wehrlin, Julien Rysman, Olivier Girard

**Affiliations:** 1 ISSUL, Institute of Sports Sciences, University of Lausanne, Lausanne, Switzerland; 2 Department of Physiology, Faculty of Biology and Medicine, University of Lausanne, Lausanne, Switzerland; 3 Swiss Federal Institute of Sport, Section for Elite Sport, Magglingen, Switzerland; 4 Faculty of Motor Sciences, Université Libre de Bruxelles, Brussels, Belgium; 5 ASPETAR, Orthopaedic and Sports Medicine Hospital, Athlete Health and Performance Research Centre, Doha, Qatar; Université Claude Bernard Lyon 1, FRANCE

## Abstract

**Purpose:**

We investigated association of hematological variables with specific fitness performance in elite team-sport players.

**Methods:**

Hemoglobin mass (Hb_mass_) was measured in 25 elite field hockey players using the optimized (2 min) CO-rebreathing method. Hemoglobin concentration ([Hb]), hematocrit and mean corpuscular hemoglobin concentration (MCHC) were analyzed in venous blood. Fitness performance evaluation included a repeated-sprint ability (RSA) test (8 x 20 m sprints, 20 s of rest) and the Yo-Yo intermittent recovery level 2 (YYIR2).

**Results:**

Hb_mass_ was largely correlated (r = 0.62, P<0.01) with YYIR2 total distance covered (YYIR2_TD_) but not with any RSA-derived parameters (r ranging from -0.06 to -0.32; all P>0.05). [Hb] and MCHC displayed moderate correlations with both YYIR2_TD_ (r = 0.44 and 0.41; both P<0.01) and RSA sprint decrement score (r = -0.41 and -0.44; both P<0.05). YYIR2_TD_ correlated with RSA best and total sprint times (r = -0.46, P<0.05 and -0.60, P<0.01; respectively), but not with RSA sprint decrement score (r = -0.19, P>0.05).

**Conclusion:**

Hb_mass_ is positively correlated with specific aerobic fitness, but not with RSA, in elite team-sport players. Additionally, the negative relationships between YYIR2 and RSA tests performance imply that different hematological mechanisms may be at play. Overall, these results indicate that these two fitness tests should not be used interchangeably as they reflect different hematological mechanisms.

## Introduction

Hemoglobin mass (Hb_mass_) represents a major convective determinant of oxygen (O_2_) supply to active muscle [1]. As such, Hb_mass_ is often regarded as a key limiting factor to maximum O_2_ uptake (V̇O_2_max), which in turn is a strong predictor of endurance performance [2]. Outside genetic predisposition [3, 4], the main influencing factors of any Hb_mass_ modification rely on training-induced adaptations [1]. Furthermore, endurance training likely impacts other hematological variables [5]: blood volume (BV) changes [5] generally outpace Hb_mass_ increase, mainly due to an exercise-induced plasma volume (PV) expansion, resulting in lower hemoglobin concentration ([Hb]) and hematocrit levels (Hct) in endurance athletes [6].

Prevention of iron deficiency plays a key role in determining fitness performance level because iron is an essential component of hemoglobin. Previous studies compared Hb_mass_, blood morphology, BV [7, 8], as well as iron metabolism [7] between endurance and power-based disciplines. From this, it results that exercise type, training workloads and duration affect the levels of the aforementioned blood indices with a more pronounced decreased iron stores in endurance sports, due to the ‘traumatic’ side-effect of running [7]. Such evaluation in team sports is scarce—to date, iron status was not associated to Hb_mass_ or to V̇O_2_max in elite field hockey players [9]–and this may relate to the diverging viewpoints in the literature [10] regarding the usefulness of hematological and iron-related variables screening of elite team-sport athletes. While some research groups have included a Hb_mass_ evaluation in their team-sport fitness test batteries—i.e., football [11] or Australian Football League [12]–this practice is not yet widely accepted. Inclusion of a Hb_mass_ evaluation as part of the regular team sports screening may bring additional insight into the underlying hematological adaptive mechanisms to training and competition.

Compared to endurance athletes [8], male team-sport players generally possess low to moderate Hb_mass_ (9–13 g.kg^-1^) or V̇O_2_max values (55–65 mL.min^-1^.kg^-1^) [4]. While close associations of Hb_mass_ and/or BV with V̇O_2_max have been widely documented in endurance disciplines [8, 13], evidence for such relationships in cohorts of team-sport players is still limited [9]. Interestingly though, Hinrichs et al. [9] reported that Hb_mass_ (12.5 ± 0.9 g.kg^-1^)–but not [Hb] and Hct—correlated positively (r = 0.57) to V̇O_2_max (55.8 ± 4.0 mL.min^-1^.kg^-1^) in field hockey players, while others demonstrated a negative correlation between Hct and aerobic capacity in footballers [14]. In this study, V̇O_2_max was determined using an incremental running protocol (+ 0.7 km.h^-1^ every 30 s with a constant gradient of 2% on a treadmill). However, the direct application of findings obtained from a continuous, laboratory-based test protocol is not straightforward in team sports [15]. Implementation of intermittent, field-based protocols such as the widely used Yo-Yo intermittent recovery (YYIR) tests [15] would add ecological validity to elucidate the association of hematological indices with aerobic performance (i.e. distance covered) specific to team sports. Although theoretical V̇O_2_max can be estimated (r = 0.58) from the YYIR2 total distance covered (YYIR2_TD_), this latter variable appears more specific to reflect the intermittent pattern of team sports [15].

Time-motion analyses [16] indicate that competitive field hockey fitness demand is high and involves the repetition of high-intensity work phases separated by periods (often incomplete) of lower-intensity recovery. Reportedly, the total distance covered by New Zealand men’s squad outfield hockey players over a 70-min match averaged 8160 m, of which high-intensity running (> 19 km.h^-1^) represented 6.1% (479 ± 108 m) and involved ~34 sprints per player; average sprint duration and mean recovery duration were 3.3 s and 125 s [ranging from < 20 s (16% of the time) to > 60 s (55%)], respectively [16]. Besides inherent variability of game characteristics in field hockey between playing positions and playing styles, it is remarkable that sprinting activities for forward players decrease by ~12% between the first and the second half [17]. In addition to the considerable aerobic demands placed on top-level field hockey players [16], the ability to repeatedly perform “all-out” efforts with incomplete recoveries (repeated-sprint ability or RSA) is considered an important fitness component of this sport [18]. To date, the nature of the relationship between hematological variables and RSA is unknown. Therefore, the aim of this study was to determine the nature of the association of different hematological parameters, mainly Hb_mass_, with sport-specific fitness performance (aerobic and RSA) in a cohort of elite field hockey players.

## Methods

The study and informed consent form were approved by the Anti-Doping Lab Qatar institutional review board (Agreement SCH-ADL-070) and conformed to the current Declaration of Helsinki guidelines. All subjects provided written informed consent.

### Subjects

Twenty-five elite male field hockey players (age 26.5 ± 4.4 years, height 178.8 ± 6.3 cm, body mass 76.2 ± 7.8 kg, BMI 23.8 ± 1.7 kg.m^-2^) were recruited among Belgium, Spanish and Dutch first division clubs (9 of the participants were national team members of their respective countries) to participate in this study. Players neither conducted an altitude training intervention in the past 6 months before testing nor reported any iron supplementation or donated blood at the time of the investigation. Typical weekly training load was 7–9 h per week and involved hockey practice and conditioning activities aiming to replicate activity patterns and physiological and neuromuscular demands of elite games.

### Procedures

As part of a screening procedure completed during the competitive outdoor in-season (i.e., January), subjects underwent the following assessments: (i) duplicate 2-min optimized carbon monoxide (CO)-rebreathing method to measure Hb_mass_ and calculate red cell (RCV), blood (BV) and plasma (PV) volumes, (ii) venous blood sampling to determine [Hb], Hct, mean corpuscular hemoglobin concentration (MCHC), serum iron, serum ferritin and transferrin, and (iii) fitness performance testing (RSA and YYIR2). Time separating (i) or (ii) and (iii) ranged between 12 h and 24 h. Subjects were asked to refrain from strenuous exercise, to avoid caffeine and alcohol in the 24 h preceding the measurements, and to arrive at the testing sessions in a rested and hydrated state, at least 3 h postprandial.

### Determination of hemoglobin mass and blood volume parameters

Hb_mass_ was measured by using a modified version by Steiner and Wehrlin [19] of the optimized carbon monoxide (CO)-rebreathing method originally described by Schmidt and Prommer [20]. Briefly, the subjects inhaled a bolus of 100 mL pure CO (Multigas SA, Domdidier, Switzerland) followed by 3.5 L O_2_ using a specific glass spirometer (Blood Tec GbR, Bayreuth, Germany) and this gas mixture was rebreathed in a closed circuit system for 2 min. Capillary blood samples (35 μl) from an earlobe were collected immediately before and 6 and 8 min after the rebreathing and analyzed for carboxyhemoglobin (ABL 800flex, Radiometer A/S, Copenhagen, Denmark). The remaining CO in the system and the end-tidal CO concentration were determined using a CO analyzer (Dräger PAC 7000; Dräger Safety; Lübeck, Germany) to calculate the amount of CO that was not taken up during the inhalation and the amount exhaled after the test. The total Hb_mass_ was calculated as previously described [20], using a slightly different correction for CO flux to myoglobin (0.3% × min^-1^ of administered CO) as recommended by Prommer & Schmidt [21]. The reliability of the CO-rebreathing method in our mobile laboratory was characterized by a typical error of 1.5%, with a derived typical error of 1.0% from the use of all duplicate measurements throughout the study. The Hb_mass_ values were expressed as the mean of the duplicate measurements and were normalized to body mass.

Further to Hb_mass_ determination, RCV, BV and PV were estimated as follows [8]:

$$\text{RCV }={\text{ Hb}}_{\text{mass}}/\text{ MCHC }\times \;100$$

BV = RCV × (100 / Hct)

PV = BV – RCV

### Venous blood sampling and analysis

Venous blood samples (9 mL; 4 mL for EDTA blood, 5 mL for blood serum) were drawn in the morning without any preceding severe exercise from an antecubital vein after a 10-min rest period in a sitting position. Aliquots of 2 mL were placed into EDTA tubes to determine [Hb], Hct (i.e. corrected to whole-body hematocrit by the cell factor 0.91), and MCHC using a CELL-DYN 3700 SL analyzer (Abbott Diagnostics, Chicago, USA) and adjusted for BV changes as previously described [22]. In addition, 5 mL of the venous blood was centrifuged, while the serum was used to measure the concentrations of serum-iron, ferritin (CMIA, Chemiluminescent Microparticle Immunoassay) and transferrin using an Architect CI8200 (Abbott Diagnostics, Chicago, USA).

### Fitness performance testing

Testing was performed inside a well-ventilated gymnasium on a synthetic ground (Taraflex^®^) at constant ambient temperature/relative humidity of ~22.0°C/55%. Subjects were familiar with all testing procedures, as part of their regular testing routine. After a standardized and supervised 15 min warm-up (i.e. athletic and acceleration drills), they performed a RSA test, followed after 15 min of recovery (i.e. seating), by a specific aerobic fitness test. They were vigorously encouraged to perform maximally during all efforts.

#### Repeated-sprint ability

The RSA test consisted of eight, 20 m straight-line maximal sprints in alternating directions interspersed by 20 s of passive recovery. Sprint times were measured to the nearest 0.01 s using photocells connected to an electronic timer (Polifemo Radio Light, Microgate, Bolzano, Italy) and placed at 0 and 20 m distance intervals. Photocells height was adjusted according to the height of the subject’s hip. Subjects were asked to assume a standing, ready position 50 cm behind the starting photocell gate for 3 s before each sprint bout. During the first sprint, the 95% criteria score (defined from the best of three single sprints recorded beforehand; data not presented) was satisfied by all subjects. RSA was assessed from sprinting times data using three scores: the best sprint time (RSA_best_), the total sprint time (i.e. sum of the eight sprints; RSA_TT_) and the sprint decrement score [RSA_Sdec_ (%) = ((RSA_TT_) / (RSA_best_ × 8)– 1) × 100] [18].

#### Specific aerobic fitness

The Yo-Yo Intermittent Recovery level 2 test (YYIR2) was conducted to assess high-intensity intermittent running performance [15]. Briefly, this incremental running test to exhaustion consists in the repetition of two 20-m runs, at progressively increased speeds (starting at 13 km.h^-1^), interspersed by 10-s active recovery periods and controlled by audio beeps. The test ended when the subjects had failed to reach the finishing line in time for the second time and the YYIR2_TD_ was then recorded. This test is reproducible and is described as a sensitive tool to differentiate between playing positions and competitive levels or assess variability of intermittent exercise performance at different seasonal periods [15].

During the YYIR2 test, heart rate (HR) was recorded at 5-s intervals using a HR monitor (S610i, Polar Electro, Kempele, Finland) with the highest value (sustained during 15 s) retained as maximal HR (HR_max_). None of the subjects reported a HR_max_ < 95% of their age-predicted HR_max_ (i.e. traditional 220-age formula), indicating maximal exhaustion.

### Statistical Analyses

All statistical calculations were made using Sigmaplot 11.0 software (Systat Software, San Jose, CA, USA). Data were initially checked for normal distribution using the Shapiro-Wilk test. Linear regressions were used to determine the Pearson’s product-moment correlation coefficients (r)–with confidence intervals set at 90% (90% CI)–between the different hematological and fitness performance variables. Magnitude of r values was considered as trivial (r < 0.1), small (0.1 < r < 0.3), moderate (0.3 < r < 0.5), large (0.5 < r < 0.7), very large (0.7 < r < 0.9), nearly perfect (r > 0.9) and perfect (r = 1.0). In a further step, multiple linear regression models (stepwise backward elimination procedure) was used successively with YYIR2_TD_, RSA_best_, RSA_TT_ and RSA_Sdec_ as the dependent variables to determine the most influencing hematological and iron-related variables for the prediction of each of these fitness performance parameters. Variables with F value < 4 were removed from the model. The r^2^ values derived from the multiple linear regression models were converted to r values to use the latter criteria to interpret the magnitude of the relationships. Results are expressed as mean value ± standard deviation (SD) or 90% CI. Significance was set as P < 0.05 for all analyses.

## Results

**Fig 1:**
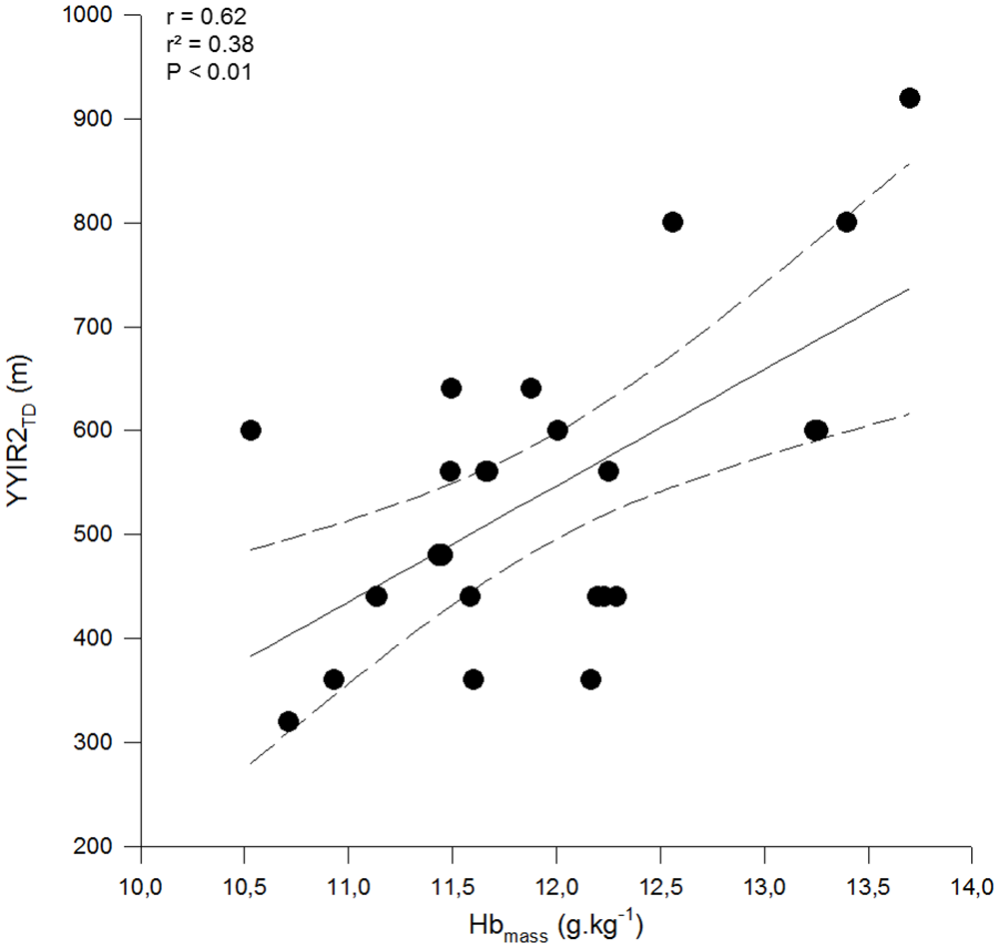
Zero-order correlations between Yo-Yo intermittent recovery test level 2 total distance covered (YYIR2_TD_, in m) and hemoglobin mass (Hb_mass_, in g.kg^-1^) in elite male (N = 25) field hockey players. Dotted lines are 95% confidence interval.

Descriptive hematological, iron-related and fitness performance parameters are presented in Table 1. Large correlations were found between Hb_mass_ and YYIR2_TD_ (r = 0.62 (90% CI 0.36; 0.79); P < 0.01) (Fig 1). [Hb] and MCHC were moderately correlated with YYIR2_TD_ (r = 0.44 (0.12; 0.68) and 0.41 (0.08; 0.66), respectively; both P < 0.05), as well as with RSA_Sdec_ (r = -0.41 (-0.66; -0.08) and -0.44 (-0.68; -0.12), respectively; both P < 0.05). Hb_mass_ did not significantly (P > 0.13) correlate with RSA_best_ (r = -0.26 (-0.55; 0.08)), RSA_TT_ (r = -0.32 (-0.59; 0.02)) and RSA_Sdec_ (r = -0.06 (-0.39; 0.28)). None of the other investigated hematological parameters or biochemical markers of iron metabolism yielded significant relationships with any fitness performance parameters (r values ranging from -0.14 (-0.46; 0.21) to 0.29 (-0.05; 0.57); all P > 0.05).

**Table 1:** Hematological parameters, biochemical markers of iron metabolism and fitness performance variables.

**Hematological parameters**						
**Hb**_**mass**_ **(g.kg** ^**-1**^ **)**	**[Hb] (g.dL** ^**-1**^ **)**	**Hct (%)**	**MCHC (g.dL** ^**-1**^ **)**	**RCV (mL)**	**BV (mL)**	**PV (mL)**
12.0 ± 0.8 (10.5–13.7)	15.2 ± 0.8 (13.9–17.4)	45.3 ± 1.9 (42.1–51.0)	33.5 ± 0.6 (32.7–34.5)	2741 ± 356 (2120–3557)	6650 ± 850 (5178–8387)	3909 ± 529 (3057–4955)
**Biochemical markers of iron metabolism**						
**Ferritin (μg.L** ^**-1**^ **)**	**Transferrin (mg.dL** ^**-1**^ **)**	**Iron (μg.dL** ^**-1**^ **)**				
137.7 ± 68.5 (45.0–279.0)	258.7 ± 39.0 (202.0–352.0)	136.4 ± 70.0 (55.0–322.0)				
**Fitness performance variables**						
**YYIR2**_**TD**_ **(m)**	**HR**_**max**_ **(bpm)**	**V̇O**_**2**_ **max (mL.min** ^**-1**^ **.kg** ^**-1**^ **)**	**RSA**_**best**_ **(s)**	**RSA**_**TT**_ **(s)**	**RSA**_**Sdec**_ **(%)**	
543 ± 159 (320–920)	187 ± 6 (181–202)	52.7 ± 2.2 (49.7–57.8)	3.26 ± 0.13 (3.05–3.50)	27.06 ± 1.02 (24.91–29.06)	3.9 ± 2.3 (0.6–11.0)	

Values are mean ± SD (range); Hb_mass_ = hemoglobin mass; [Hb] = hemoglobin concentration; Hct = hematocrit; MCHC = mean corpuscular hemoglobin concentration; RCV = Red cell (erythrocyte) volume; BV = blood volume; PV = plasma volume; YYIR2_TD_ = distance covered during the Yo-Yo Intermittent recovery test level 2; HR_max_ = maximal heat rate; V̇O_2_max = theoretical maximal oxygen uptake (using equation from Bangsbo et al. (2008)); RSA = repeated-sprint ability; RSA_best_ = best sprint time; RSA_TT_ = total sprint time; RSA_Sdec_ = sprint decrement score.

**Fig 2:**
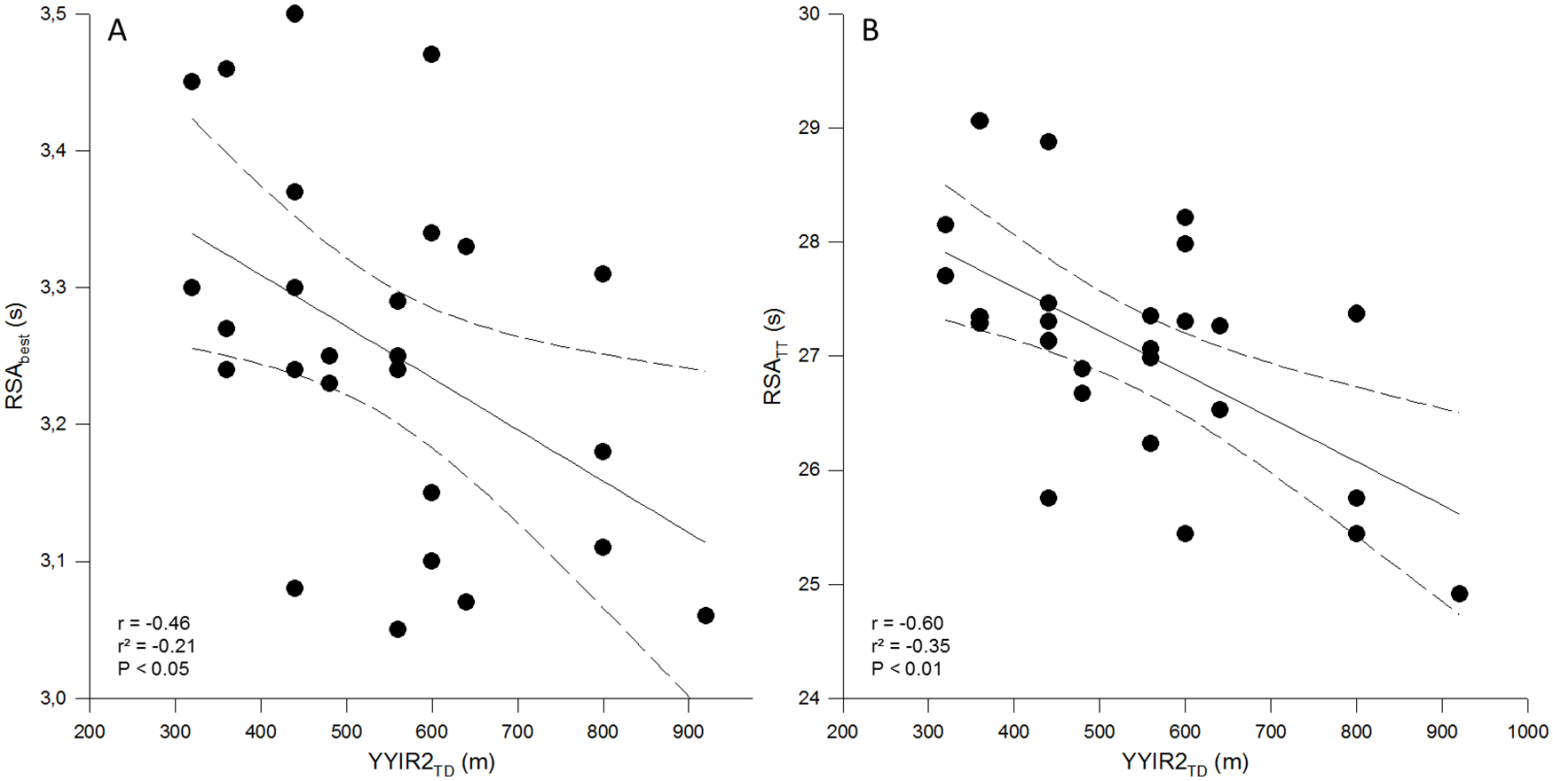
Zero-order correlations between repeated-sprint ability (best sprint time (RSA_best_), panel A and total sprint time (RSA_TT_), panel B, respectively) and Yo-Yo intermittent recovery test level 2 total distance covered (YYIR2_TD_, in m) in elite male (N = 25) field hockey players. Dotted lines are 95% confidence interval.

YYIR2_TD_ moderately correlated with RSA_best_ (r = -0.46 (-0.69; -0.15); P < 0.05) and largely correlated with RSA_TT_ (r = -0.60 (-0.76; -0.29); P < 0.01) (Fig 2), but not with RSA_Sdec_ (both r = -0.19 (-0.50; 0.16); P > 0.05).

The multiple linear regression analysis showed that only Hb_mass_ (r^2^ = 0.39, r = 0.62 (0.36; 0.79); P = 0.002) accounted significantly for the prediction of YYIR2 performance (Table 2, model 1). With RSA_best_, RSA_TT_ and RSA_Sdec_ as the dependent variables (Table 2, models 2, 3 and 4; respectively), [Hb] appeared to be the most relevant hematological predictor (P < 0.05).

**Table 2:** Hematological determinants of specific aerobic fitness and indices of repeated-sprint ability.

**Stepwise multiple regression model 1 –*YYIR2*_*TD*_**				
**Variables**	**Coefficient**	***P*** *	***r*** ^**2**^	***R***
**Intercept**	-885.37	0.002	0.39	0.62 (0.36; 0.79)
**Hb_mass_**	119.39	0.002		
**Stepwise multiple regression model 2 –*RSA*_*best*_**				
**Variables**	**Coefficient**	***P***	***r*** ^**2**^	***R***
**Intercept**	3.195	0.031	0.37	0.60 (0.34; 0.78)
**Hb**	0.267	0.018		
**Hct**	-0.082	0.044		
**Stepwise multiple regression model 3 –*RSA*_*TT*_**				
**Variables**	**Coefficient**	***P***	***r*** ^**2**^	***R***
**Intercept**	-632.51	0.098	0.28	0.53 (0.24; 0.73)
**Hb**	-43.55	0.021		
**Hct**	14.53	0.022		
**MCHC**	19.77	0.021		
**Stepwise multiple regression model 4 –*RSA*_*Sdec*_**				
**Variables**	**Coefficient**	***P***	***r*** ^**2**^	***R***
**Intercept**	22.08	0.036	0.19	0.44 (0.13; 0.67)
**Hb**	-1.184	0.059		

Coefficient of determination (*r* ^2^, stepwise multiple regression analysis) illustrating the relationships between Yo-Yo intermittent recovery test level 2 (YYIR2_TD_, model 1) or repeated-sprint ability (best sprint time (RSA_best_), model 2; total sprint time (RSA_TT_), model 3; and sprint decrement score (RSA_Sdec_), model 4) and selected hematological indices; Pearson’s *r* (90% confidence interval) was also calculated.

* *P* values denote the level of significance.

## Discussion

This study examined the relationships between hematological variables—Hb_mass_, blood morphology and volumes, as well as iron status—and sport-specific fitness performance in team-sports athletes. The main finding is that Hb_mass_ was positively correlated with specific aerobic fitness, but not with RSA in elite field hockey players. Meanwhile, moderate correlations were found between [Hb] or MCHC and RSA_Sdec_ or YYIR2_TD_. In addition, there was a negative relationship for performance between YYIR2 and RSA tests.

Hb_mass_, but not [Hb], has been previously reported to be closely related to aerobic performance (V̇O_2_max) in elite field hockey players [9]. However, aerobic evaluation in the above study was based on an incremental laboratory test, which lacks ecological validity in team-sport athletes. By using a team-sport specific aerobic field test (i.e. YYIR2), we showed that Hb_mass_ (P < 0.01), [Hb] and MCHC (both P < 0.05) were moderately to largely correlated with YYIR2_TD_ in elite field hockey players. However, differently from Heinicke et al. [8] (r ranging from 0.27 for elite swimmers to 0.67 for elite middle and long-distance runners), small correlation coefficient (r = 0.16 only) was observed here between BV and YYIR2_TD_. In addition, the moderate association of [Hb] and YYIR2_TD_ contrasts with previous findings [9, 23]. Based on Fick’s equation (or Hagen-Poiseuille law), BV (which modulates cardiac output) and [Hb] are considered as the dominant factors as they determine the O_2_ carrying-capacity and then the arterio-venous O_2_ difference [1, 8]. Whereas BV is the sum of PV and RCV, [Hb] is dependent upon the total circulating Hb_mass_ and PV. In this context, Hb_mass_ largely determines O_2_ carrying-capacity (via erythrocytosis or erythropoiesis, achieved through (i) endurance training, (ii) adaptation to altitude or (iii) blood manipulation [1]). This is clearly illustrated by its stronger relationship with sport-specific aerobic performance (i.e. YYIR2_TD_) compared with [Hb], MCHC or BV, which corroborates previous researches conducted on endurance athletes [5]. In team sports, Hb_mass_ is probably a more relevant marker of O_2_ carrying-capacity than [Hb] and would merit inclusion in routine hematological screening [11, 12] in order to bring clearer understanding of its relationships to performance as well as to doping procedures.

The hematological values (Table 1) characterizing our field hockey population including national team members are in good agreement with those for Hb_mass_ (9–13 g.kg^-1^), [Hb] (15.0–16.4 g.dL^-1^), Hct (43.9–48.1%) and V̇O_2_max (55–65 mL.min^-1^.kg^-1^) values in athletes deemed to be of similar level engaged in other team- (i.e. football, Australian Football League, handball, field- and ice-hockey) and racquet-sport (i.e. tennis, squash) disciplines [4, 7, 9, 12]. Conversely, Hb_mass_ (~14.6–15.7 g.kg^-1^), RCV (2927–3364 mL), BV (7180–7844 mL), PV (4253–4480 mL) and V̇O_2_max (~67.4–76.3 mL.min^-1^.kg^-1^) values commonly reported for elite endurance athletes (i.e., swimming, rowing, middle- and long-distance running, triathlon and cycling) are higher [8, 13], whereas [Hb] and Hct levels are comparable (15.5–15.7 g.dL^-1^ and 44.8–47.1%, respectively) [8, 13].

The mean values of ferritin, transferrin and serum-iron of tested players are in the standard range (ferritin: 15–200 μg.L^-1^; transferrin: 200–400 mg.dL^-1^; iron: 60–160 μg.dL^-1^) and none of our players was iron deficient. Furthermore, none of the investigated iron status parameters yielded significant correlations with Hb_mass_, hematological indices, BV or fitness performances. Taken as a whole, this indicates that hemoglobin values and BV are not influenced by team-sport players’ iron status when it stays in the normal physiological range (i.e. no anemia observed) [8].

To date, whether different training type, intensities and levels of performance have a singular influence on hematological indices remains unclear. The present hematological results might bring insights into the different training background between endurance and team-sport athletes. Reportedly, moderate endurance training could lead to a PV increase [6], via a ‘hemoconcentration’ phenomenon [24]. A higher PV potentially enhances exercise capacity due to increased cardiac output and a reduced blood viscosity, thereby optimizing microcirculation and improving O_2_ delivery to the working muscles [25] as well as thermoregulation [26]. While higher exercise intensities may decrease PV [27], rising training intensity by 15% over a 2-wk period would also minor [Hb] and Hct levels [28]. Altogether, the markedly lower Hb_mass_ and blood volumes in our elite field hockey players when compared to values commonly characterizing endurance athletes would suggest that training volume rather than training intensity is primarily associated with enhanced O_2_ transport capacity. It is well-known that the relationship between hematocrit (or erythrocyte) and aerobic performance is not linear but describes a bell-shaped curve with lower performance in the highest Hct distribution quintile due to hyperviscosity [14]. Although never described specifically in team-sport players, this issue is not trivial and is supported by a large body of literature (for detail, see [29–31]). It has been postulated that the influence of viscosity factors in exercise physiology should not be interpreted with the Hagen-Poiseuille law; this issue is probably much more complex, explaining to a certain extent how subjects can cope with supraphysiologic erythrocythemia. This non-linearity of viscosity factors on blood flow has been recently proposed [32, 33] to explain the ‘paradox of hematocrit’ [14], where overtraining is usually accompanied by higher [Hb]. In this view, Gaudard et al. [34] confirmed that elevated fitness is physiologically associated with a low Hct.

Aerobic metabolism during a RSA test can contribute as much as 40% of the total energy supply with V̇O_2_max being eventually reached [18]. This is, for instance, supported by previously reported correlations between V̇O_2_max and indices of RSA fatigue resistance (e.g. RSA_Sdec_ or fatigue index) [35]. Because our results also demonstrated significant association of YYIR2_TD_ with either RSA_best_ or RSA_TT_, it seems likely that “V̇O_2_max training” (i.e., high-intensity interval (e.g. 3–12 × 2 min at 110–130% V̇O_2_max with 1 min of rest) or intermittent (e.g. 12–24 × 15 s at 105–115% V̇O_2_max with 15 s of rest) workouts) is a useful intervention to improve RSA performance [36]. However, any potential improvement in blood O_2_ carrying-capacity has to be balanced in a team-sport population, so as not to limit explosive-type performance gains [37]. This assumption is strengthened by the lack of significant relationship between Hb_mass_ and any RSA parameters in our tested population. Moreover, moderate relationships between [Hb] or MCHC and RSA_Sdec_ would support the notion that other physiological systems (e.g. pH and H+ buffering role of [Hb] [38] and/or O_2_-independent substrate and/or enzyme factors implicated in maximal mitochondrial respiration rates [39]) would also determine high-intensity intermittent exercise performance by preventing excessive muscle fatigue development [8].

Finally, moderate to large correlations were seen between performance on YYIR2 and RSA tests confirming recent findings [40]. Noteworthy, such relationships were observed here despite sprinting distances (8 × 20 m with 20 s of rest) being considerably shorter when compared to the study by Ingebrigtsen et al. [40] (i.e. 7 × 35 m with 25 s of rest). Nonetheless, performance during our RSA test is probably more reliant on phosphocreatine degradation and muscle buffer capacity [41]. Although V̇O_2_max might not be the dominant determinant of RSA [35] the aerobic system contribution increases along with sprinting distance and sprint numbers in RSA protocols [18, 36, 40]. Significant correlations between YYIR2 and RSA indices may relate to the fact that both forms of exercise require repeated accelerations with short recovery between efforts. Furthermore, “RSA training” [36], as an integral part of regular training routines of our field hockey players, might have induced long-term specific adaptations (i.e., improved acceleration capacity, increased BV and clearance of lactate), in turn positively influencing intermittent running performance. Conversely to others [40], and based on different association of blood morphological and volume indices with short distance RSA and YYIR2 tests, this would indicate that these two fitness tests should not be used interchangeably as they reflect mechanisms that are likely different. This reinforces the usefulness of the approach including both YYIR and RSA tests (similar to those performed in this study) in a screening procedure of team-sport athletes.

As possible limitation, one may question if the sample size (n = 25) is large enough for a study that is based mostly on simple and multivariate correlation analyses. From a power analysis perspective (P = 0.8 and α = 0.05), 43 subjects would be needed for a multiple regression with ~10 independent factors, but only 14 subjects with simply one specific predictor. From an accuracy analysis perspective (effect size = 0.7) 14 to 35 subjects would be required, depending on the number of factors considered. An optimal experimental design would include an adequate sample size from both power and accuracy perspectives. That said, it has been demonstrated that (multi-) factorial analysis can be legitimately conducted even on similar small sample size, i.e. n ~20–30 [42–44], as in the present study. This is further supported by the a-posteriori power value > 0.8. We further consider that the elite standard of the players and the realistic testing setting (physical performance) maximize the ecological validity of the present investigation. One may also argue that the stepwise analysis includes parameters (e.g. Hb, Hct, MCHC) likely related to each other and therefore not independent predictors of performance during field tests in the models. In the present study, collinearity was carefully controlled and the independent variables systematically removed from the regression model. In addition, we tested the model’s normality (Shapiro-Wilk), constant variance and statistical power. In all cases, normality and heteroscedasticity conditions were accepted, while power reached values > 0.8. Finally, the variance inflation factor (ranging between 1.23 and 1.59) in all backward stepwise regressions has been recalculated. We are therefore confident that our backward stepwise regressions were performed without including redundant independent variables.

## Conclusion

In summary, Hb_mass_ and sport-specific aerobic fitness displayed a large positive correlation in elite male field hockey players. Meanwhile, Hb_mass_ was not associated with RSA outcomes, whereas moderate correlations were found between [Hb] or MCHC and RSA fatigue resistance indices. Additionally, the negative relationships for performance between YYIR2 and RSA tests imply that different hematological mechanisms may be at play. Further studies, where both fitness performance and hematological parameters are screened regularly during a typical team-sport season (longitudinal effect), as well as after specific- (repeated sprinting, high-intensity interval training) or hypoxic-training routine, are necessary to deepen our knowledge regarding the association between these variables and underlying mechanisms.
